# A Novel Model for Simultaneous Evaluation of Hyperoxia-Mediated Brain and Lung Injury in Neonatal Rats

**DOI:** 10.3390/cells14060443

**Published:** 2025-03-16

**Authors:** Stefanie Obst, Meray Serdar, Josephine Herz, Karina Kempe, Meriem Assili, Mandana Rizazad, Dharmesh Hirani, Miguel A. Alejandre Alcazar, Stefanie Endesfelder, Marius A. Möbius, Mario Rüdiger, Ursula Felderhoff-Müser, Ivo Bendix

**Affiliations:** 1 Department of Paediatrics I, Neonatology and Experimental Perinatal Neurosciences, Centre for Translational Neuro- and Behavioural Sciences (C-TNBS), University Hospital Essen, University Duisburg-Essen, 45147 Essen, Germany; 2 Institute for Lung Health (ILH), Cardiopulmonary Institute (CPI), Member of the German Centre for Lung Research, University of Giessen and Marburg Lung Center, 35392 Giessen, Germany; 3 Translationale Experimental Pediatrics, Department of Pediatric and Adolescent Medicine, University of Cologne, 50937 Cologne, Germany; 4 Cologne Excellence Cluster for Stress Responses in Ageing-Associated Diseases (CECAD) and Center for Molecular Medicine Cologne (CMMC), University of Cologne, 50931 Cologne, Germany; 5 Department of Neonatology, Charité-Universitätsmedizin Berlin, 13353 Berlin, Germany; 6 Department for Neonatology and Pediatric Intensive Care, Clinic for Pediatric and Adolescence Medicine, Faculty of Medicine, Technische Universität Dresden, 01307 Dresden, Germany; 7 Saxony Center for Feto-Neonatal Health, Faculty of Medicine, Technische Universität Dresden, 01307 Dresden, Germany

**Keywords:** hyperoxia-mediated lung and brain injury, preterm birth, encephalopathy of prematurity (EoP), bronchopulmonary dysplasia (BPD), myelination, vascularisation

## Abstract

Despite improved neonatal intensive care, the risk of premature-born infants developing bronchopulmonary dysplasia (BPD) and encephalopathy of prematurity (EoP) remains high. With hyperoxia being a major underlying factor, both preterm-birth-related complications are suggested to be closely interrelated. However, experimental models are lacking for the assessment of the potentially close interplay between both organs. To establish a model, suitable for the assessment of both affected organs, Wistar rats were exposed to 80% oxygen from postnatal day 2 (P2) for seven days. Brain and lung tissues were analysed via histomorphometry, immunohistochemistry, real-time PCR, and western blot at term P11. In the brain, hyperoxia induced significant hypomyelination accompanied by a reduction in oligodendrocytes and CD68 expression on microglia cells. These changes correlate with arrested alveolarisation and an increased number of macrophages in the lung. Interestingly, in contrast to the reduced formation of pulmonary microvessels, an increased vascular density was detected in the brain. Seven days of hyperoxia induces typical characteristics of BPD and EoP in neonatal rats, thereby linking impaired alveolarisation with disturbed myelination in the brain and providing an experimental model for understanding pathophysiological mechanisms and identifying organ-spanning novel therapeutic interventions targeting both diseases.

## 1. Introduction

Affecting 10% of all newborns worldwide, prematurity is the leading cause of child mortality and morbidity [1]. Improvements in neonatal intensive care increased the survival rates of extremely preterm-born infants (<28 weeks of gestation) with reduced morbidity rates of retinopathy of prematurity and late-onset sepsis [2,3,4]. However, other long-term sequelae such as encephalopathy of prematurity (EoP) and the chronic lung disease bronchopulmonary dysplasia (BPD) remained high and increased, respectively [5,6,7,8]. Compared to in utero conditions, infants are exposed to three-fold higher oxygen levels in the atmosphere, suggesting relative hyperoxia as the major cause of BPD and EoP in preterm infants [9,10]. Due to immature antioxidant defence mechanisms in preterm infants, immature organs are unable to cope with hyperoxia-induced oxidative stress [11,12,13]. In addition, life-saving medical interventions such as supplemental oxygen and ventilation may further exacerbate the detrimental effects of increased oxygen concentrations relative to in utero conditions [14,15]. Although careful ventilation methods have been established, optimal strategies to reduce the incidence of hyperoxia-mediated BPD and EoP are missing [11,16,17,18,19,20]. Furthermore, infants with BPD are at higher risk for adverse neurodevelopmental outcome [21,22].

Over the last few decades, experimental studies have used various hyperoxia-mediated lung and brain injury models to analyse underlying pathomechanisms and test therapeutic approaches. However, the timing and duration of hyperoxia widely varied between these BPD and EoP models [23]. To model a BPD phenotype, hyperoxia is commonly initiated around birth with oxygen concentrations higher than 85% for approximately 14 days [24,25,26]. In contrast, hyperoxia-based models of EoP used much shorter exposure (6–48 h) with lower oxygen concentration (80%) and were initiated later (starting from postnatal day 3 (P3) to P6) [27,28,29,30]. Hyperoxia disturbs oligodendrocyte differentiation and increases degeneration of oligodendrocyte progenitor cells, leading to hypomyelination in EoP models [28,31,32,33], which is associated with adverse long-term neurological outcome such as cognitive and neuropsychiatric disabilities [34,35]. In BPD models, hyperoxia leads to an arrest of alveolar development, resulting in long-term impairment of lung structure and respiratory function [36,37]. Besides hypoalveolarisation, BPD is associated with reduced vascular density [38], whereas effects on vascularisation in the developing brain are largely unknown. Because of similarities in inflammatory and oxidative stress responses in both organs [23], we hypothesised a detrimental impact of hyperoxia on vascularisation in the brain, comparable to reduced pulmonary vascular architecture. Up to now, both organs have been rarely assessed in the same experimental model. Only a few studies investigated brain tissues in BPD models, revealing detrimental impacts of up to 14 days of hyperoxia on neurovascular coupling, neurogenesis, and inflammation [39,40,41,42,43,44]. However, the potential impact on white matter development, particularly important for long-term neurological function, is not well studied. Furthermore, BPD models use a prolonged hyperoxia duration of up to 28 days in rodents, which corresponds to toddlers, when translated to human development [45]. Therefore, we established a model with a shorter duration of hyperoxia, corresponding to neonatal development covering critical phases of both organs simultaneously.

In the present study, we hypothesised that seven days of hyperoxia affect both brain and lung development, mimicking effects typically seen in EoP and BPD models. Following hyperoxia starting at P2, we analysed its impact on oligodendrocyte maturation, vascularisation, and microglia activation in the brain and simultaneously evaluated alveolarisation, vascularisation, and macrophage infiltration in developing lungs at P11, i.e., term-equivalent age.

## 2. Materials and Methods

### 2.1. Animals and Experimental Procedure

All animal experiments were approved and performed in accordance to guidelines of the University Hospital Essen, Germany, and with local government approval by the State Agency for Nature, Environment and Consumer Protection North Rhine–Westphalia. Animals were housed under 12 h light/dark cycle, with food and water ad libitum. Two-day-old (P2) Wistar rat pups were placed in an oxygen chamber containing 80% oxygen (OxyCycler, BioSpherix, Parish, NY, USA; 80% O_2_, HO) together with their lactating dams for seven consecutive days. Control animals were maintained under normoxic conditions in room air (21% O_2_, NO). Dams were changed daily between room air and hyperoxia to avoid respiratory distress. The pups’ body weight was determined daily from postnatal day 0 (day of birth (P0)) to P11, showing no differences between both groups (NO: 24.4 g ± 2.4 g, HO: 23.3 g ± 2.7 g). Pups were sacrificed at P11, under deep anaesthesia with chloral hydrate (200 mg/kg body weight in max. 0.1 mL injection volume/10 g body weight). This time point corresponds to brain development of a term-born infant and the alveolar phase of lung development [23]. For pulmonary gene and protein analysis, the right lung lobe was tied off with a surgical thread, dissected, and snap-frozen in liquid nitrogen. The left lung lobe was intratracheally pressure-fixed at 20 cm H_2_O with 4% paraformaldehyde (PFA) for 5 min. During lung inflation, animals were perfused transcardially with ice-cold phosphate-buffered saline (PBS). After inflation, the trachea was tied, and the inflated left lung lobe was dissected with the heart. After an immersion fixation in 4% PFA for at least 24 h at 4 °C, the left lobe was dehydrated and embedded in paraffin. The brain was removed, and the hemispheres were separated. The right hemisphere was fixed in 4% PFA for at least 24 h at 4 °C, dehydrated, and embedded in paraffin, whereas the left hemisphere was snap-frozen on dry ice. A total number of 26 (13 female and 13 male) rat pups were included in this study and randomly assigned to the treatment groups. No animals died during the study.

### 2.2. Lung Histomorphometric Analysis

Paraffin-embedded lungs were cut at random angles in two perpendicular planes for isotropic uniform random (IUR) orientation, using a randomiser to select the angles, and were remounted on the embedding cassette for sectioning [46]. To analyse the morphological structure of the lung, random lung sections were stained with haematoxylin and eosin. Briefly, after deparaffinisation and rehydration, lung tissue sections were stained with haematoxylin for 5 min, followed by washing under running tap water, counterstaining with eosin for 8 min, and dehydration. Using the 40× objective, large-scale images were acquired with a Leica Aperio ScanScope scanner (Leica Biosystems, Wetzlar, Germany). Up to 10 random non-overlapping fields of view (397.254 µm^2^) were generated with the Aperio Image Scope software (version 12, Leica Biosystems, Germany). Images with non-inflated and atelectatic regions were excluded from the morphological analysis. Alveolar diameter and septal thickness were assessed in 10 randomly selected alveoli per field of view (FOV) using ImageJ software (1.54, National Institutes of Health, Maryland, MD, USA). The average surface of an alveolus was calculated with the following formula: Surface area = 4 × π × r^2^. Septal thickness was measured from the inner to the outer surface of the alveolar septum. The mean linear intercept was analysed using Cell D 3.4 Olympus soft image solutions (Olympus, Hamburg, Germany). Briefly, a grid of lines was used to count the intercepts of lines and alveolar walls, and the total number of interceptions per field was divided by the total length of the lines [46]. Due to incomplete inflation of the lungs, three hyperoxia-treated animals were excluded from morphological analyses.

### 2.3. Brain and Lung Immunohistochemistry

Immunohistochemical analysis was performed on 7 µm paraffin and 20 µm cryostat brain sections at the hippocampal level −3.72 ± 0.7 mm from bregma and on randomly chosen sections of the lung. Paraffin sections were deparaffinised and rehydrated, and antigen-retrieval was performed in pre-heated sodium citrate buffer (10 mM tri-sodium citrate, 0.05% Tween-20; pH 6.0) at 100 °C for 30 min. Unspecific antibody binding was blocked with 1% bovine serum albumin (BSA), 0.3% coldfish skin gelatine (Sigma-Aldrich, St. Louis, MO, USA), and 0.2% Tween-20 in PBS (brain sections) or Tris-buffered saline (TBS) (lung sections) for 1 h, followed by primary antibody incubation overnight at 4 °C.

Hypomyelination was analysed on brain tissue sections stained for myelin basic protein (MBP). The effect of hyperoxia on the overall number of oligodendrocytes and their maturation was investigated by co-staining the pan-oligodendrocyte marker oligodendrocyte transcription factor 2 (OLIG2) and adenomatous polyposis coli, clone CC1 (CC1). Microglia activation and morphology were determined by co-staining ionised calcium-binding adaptor protein-1 (IBA1) and macrosialin (CD68, cluster of differentiation 68). Vascular and oligodendrocyte progenitor cells were analysed on frozen native cryostat brain slices. Briefly, sections were thawed and dried at 37 °C and room temperature, each for 30 min followed by fixation in ice-cold acetone/methanol (1:1). Unspecific antibody binding was blocked with Protein Block Serum-Free (Agilent DAKO, Santa Clara, CA, USA) followed by incubation with anti-vWF (von Willebrand factor) antibody or the double staining of OLIG2 and platelet-derived growth factor receptor alpha (PDGFRA) overnight at 4 °C. Lung tissue sections were analysed for macrophage infiltration by staining CD68, and vessel densities were determined by staining vWF. Antibody binding was visualised by incubation with appropriate anti-rat/mouse/rabbit Alexa Flour 488, Alexa Flour 555, and Alexa Flour 647 conjugated secondary antibodies (all 1:500, Thermo Fisher Scientific, Waltham, MA, USA) for 1 h at room temperature. Sections were counterstained with 4′,6-diamidino-2-phenylindole (DAPI) (1 μg/mL, Invitrogen, Carlsberg, CA, USA). Detailed information on primary antibodies is provided in Table S1.

### 2.4. Confocal Microscopy

Stained brain and lung sections were analysed by confocal microscopy (A1plus, Eclipse Ti, with NIS Elements AR 5.30.00 software, Nikon, Tokyo, Japan) using 10× and 20× objectives. Four laser lines (laser diode: 405 nm; Ar laser: 514 nm; G-HeNe laser: 543 nm; Rn laser: 639 nm) and three different filters (450/50-405 LP, 515/20-540 LP, and 585/65-640 LP) were used for image acquisition. Confocal *z*-stacks of 5 µm thickness (*z*-plane distance 1 µm) for paraffin brain and lung sections and 14 µm thickness for cryostat brain sections (*z*-plane distance 2 µm) were acquired. Confocal *z*-stacks were converted into 2-dimensional images using maximum intensity projection. Observers performing image acquisition and analysis were blinded to experimental conditions. Quantification was performed with the NIS Elements AR 5.30.00 software (Nikon) in two sections per animal. Fields of view (FOV) used for quantification are shown in Figure S1. 

Myelination analysis was performed using large-scale images of the whole right hemisphere. The percentage of the MBP-positive area and the sum pixel intensity (sum of the intensity of every pixel) were determined. The number of oligodendrocytes was quantified in three images (302.5 µm^2^ each) of the cortex and the thalamus, as well as in four images of the white matter (cingulum, deep cortical, external, and internal capsule) (Figure S1). For microglia analysis, four regions within the white matter (302.5 µm^2^ each) were analysed. The percentage of the total IBA1^+^ area, cell circularity of IBA1^+^ cells, and the percentage of the CD68^+^ area from the total IBA1^+^ area were measured. Vascular density was investigated by the quantification of the percentage of vWF-positive area and sum pixel intensities in four brain regions (cortex, white matter, hippocampus, and thalamus, Figure S1).

Macrophage infiltration and vessel density on lung tissues were evaluated in 10 random non-overlapping images (187.9 µm^2^ each). CD68^+^ cells were counted, and CD68^+^ cells/mm^2^ were calculated. For vascular analysis, vessels per FOV were counted, and the maximum diameter of each vessel was measured from the outer vessel walls. Longitudinal cut vessels were excluded, as the maximum diameter could not be determined. The distribution of vessel diameter was examined using a histogram (diameter versus number of vessels). Subsequently, vessel density (number of vessels/FOV) was analysed for different categories according to the vessel diameter (all vessels, less than 20 µm, 20–40 µm, and 40–60 µm).

### 2.5. Immunoblotting

Protein isolation and blotting were performed as described previously [28,31] without the fractional isolation of the mitochondrial and nuclear fraction. Briefly, the protein was isolated from a 640 µm thick section of the hippocampal level—4.74 ± 0.3 mm from bregma and half of the right lung lobe. Brain tissue was lysed in Radio-Immunoprecipitation Assay (RIPA) buffer (Sigma-Aldrich) with phosphatase inhibitors (Roche, Basel, Schwitzerland) by up-and-down pipetting. Lung tissue was mechanically homogenised in RIPA buffer using a steel ball per sample and the tissue lyser (Qiagen, Hilden, Germany). Subsequently, both tissue homogenates were incubated for 20 min on ice, followed by centrifugation at 17,000× *g*. Cytosolic supernatants were transferred into fresh reaction tubes and stored at −80 °C until further use. Protein concentration was quantified using a BCA kit (Thermo Fisher Scientific, Dreieich, Germany) and adjusted to 4 µg/µL with 2× Laemmli buffer and distilled water. Laemmli samples were denatured for 10 min at 95 °C and stored at −20 °C for up to two weeks until gel electrophoresis. 40 µg protein per lane was separated in 10% and 12.5% polyacrylamide gels including 2,2,2-trichlorethanol for visualisation of total protein abundance and transferred to nitrocellulose membranes (0.2 μm Amersham, Cytiva, Markborough, MA, USA) at 4 °C overnight using a tank blotting system (Bio-Rad, Herkules, CA, USA). Equal loading and transfer of proteins were confirmed with ultraviolet light-activated photoreactive shift in tryptophan residues with 2,2,2-trichlorethanol [47]. Fluorescent bands of the total protein on the membrane were captured using the ChemiDoc Imaging System (Bio-Rad). Non-specific binding sites were blocked with 5% BSA (Carl Roth, Karlsruhe, Germany), 0.1% Tween-20 in TBS (TBS-T) (neural glial antigen 2 (NG2)) or 5% non-fat milk powder (Cell Signaling, Danvers, MA, USA), 0.1% Tween-20 in TBS (all other antibodies). Membranes were incubated at 4 °C overnight with the following primary antibodies: mouse anti-MBP, mouse anti-2′,3′-cyclic-nucleotide 3′-phosphodiesterase (CNPase), mouse anti-myelin-associated glycoprotein (MAG), rabbit anti-NG2, rabbit anti-OLIG2, and rabbit anti-IBA1 (Table S2). After incubation with appropriate peroxidase-conjugated secondary antibodies in TBS-T (1:5000 for appropriate anti-mouse isotype (Novus Biologicals, Littleton, CO, USA); 1:2000 for anti-rabbit (Cell Signaling)), antibody binding was detected by using enhanced chemiluminescence (GE Healthcare Life Sciences, München, Germany). Densitometric analysis was performed with ImageLab 6.1 software (Bio-Rad). Proteins of interest were normalised to the total protein, and reference blots are shown in the supplement (Figure S2).

### 2.6. Real-Time PCR

Gene expression analysis was performed as described previously using Taqman Real-Time PCR Assays (Thermo Fisher Scientific) [31]. Briefly, for brain mRNA analysis, a 640 µm thick tissue section was collected at the hippocampal level −5.34 ± 0.3 mm from bregma of the left hemisphere. RNA was isolated with an RNeasy Mini Kit and DNase I treatment (Qiagen, Germany). For lung gene expression, mRNA of the other half of the right lobe was isolated with TRIzol/chloroform extraction using the QIAzol reagent (Qiagen) and DNase I treatment (Qiagen). Briefly, 1.6 µg of RNA was reversed-transcribed using SuperScript II Reverse Transcriptase (Invitrogen). TaqMan real-time PCR was performed in duplicates in 96-well optical reaction plates for 40 cycles, with each cycle at 94 °C for 15 s and 60 °C for 1 min using the StepOne-Plus Real-Time PCR system (Applied Biosystems/Thermo Fisher Scientific, Waltham, WA, USA). PCR products were quantified using assay-on-demand primers and TaqMan^TM^ Fast Advanced Master Mix (Applied Biosystems/Thermo Fisher Scientific, Table S3). Ct values were normalised to the housekeeping gene beta-2-microglobulin (B2M) [Δct = ct (target gene) − ct (beta-2-microglobulin)] and related to the mean of control animals using the 2^−ΔΔCT^ formula [48]. Fold change values were calculated.

### 2.7. Statistical Analysis

Statistical analysis was performed with GraphPad Prism 6 (GraphPad Software, Boston, MA, USA). Data were tested for Gaussian distribution and analysed either by Student’s *t*-test (parametric) or Mann–Whitney U test (non-parametric), respectively. Data are presented in scatter plots as mean ± standard deviation (SD). *p*-values less than 0.05 were considered statistically significant.

## 3. Results

### 3.1. Seven Days of Hyperoxia Induce Hypomyelination in the Immature Brain

**Figure 1:**
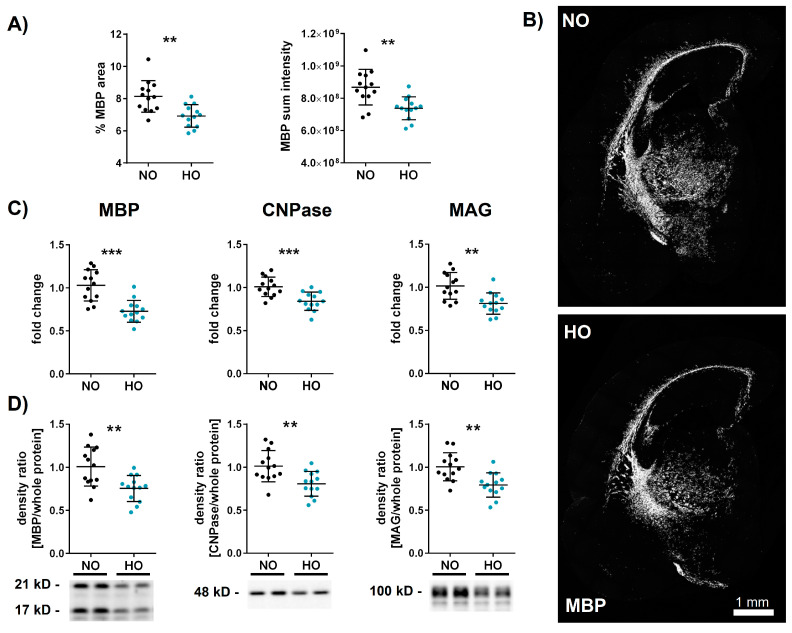
Seven days of hyperoxia leads to myelination deficits in the immature brain. Neonatal Wistar rats were exposed to 80% oxygen from postnatal day 2 (P2) to P9. Brains were analysed on P11, and the percent MBP-positive area and sum pixel intensity (**A**) were evaluated in the whole hemisphere of hippocampal brain sections (scale bar 1 mm; (**B**)). Hypomyelination was confirmed with real-time PCR (**C**) and western blot (**D**) analysis of MBP, CNPase, and MAG. Proteins of interest were normalised to the total protein, and reference blots are shown in the supplement (Figure S2). Data are presented in scatter plots with mean ± standard deviation; *n* = 13 animals/group; Student’s *t*-test: ** *p* < 0.01, *** *p* < 0.001.

To investigate the effect of hyperoxia (HO) on the immature brain and lung in one experimental model, neonatal Wistar rats were exposed to 80% oxygen for seven days beginning at postnatal day 2 (P2). Control animals remained under room air/normoxia for the same time period (21% O_2_, NO). Analyses were performed at P11, which equals the brain development of a term-born human infant [49]. A key feature of EoP is hypomyelination, which can be experimentally induced by postnatal hyperoxia [9,44]. To assess effects of hyperoxia on myelination, hippocampal brain sections were stained for MBP (Figure 1A,B). Compared to controls, hyperoxia-treated animals showed a reduced MBP-positive area and sum pixel intensity (Figure 1A). Besides MBP, other myelin-associated proteins like 2′,3′-cyclic-nucleotide 3′-phosphodiesterase (CNPase) and myelin-associated glycoprotein (MAG) are important for a correct formation of the myelin sheet. MBP, CNPase, and MAG revealed a declined expression in the hippocampal level in the hyperoxia group as assessed by real-time PCR and Western blot analysis (Figure 1C,D).

### 3.2. Hyperoxia Reduces the Number of Oligodendrocytes in the Developing Brain

**Figure 2:**
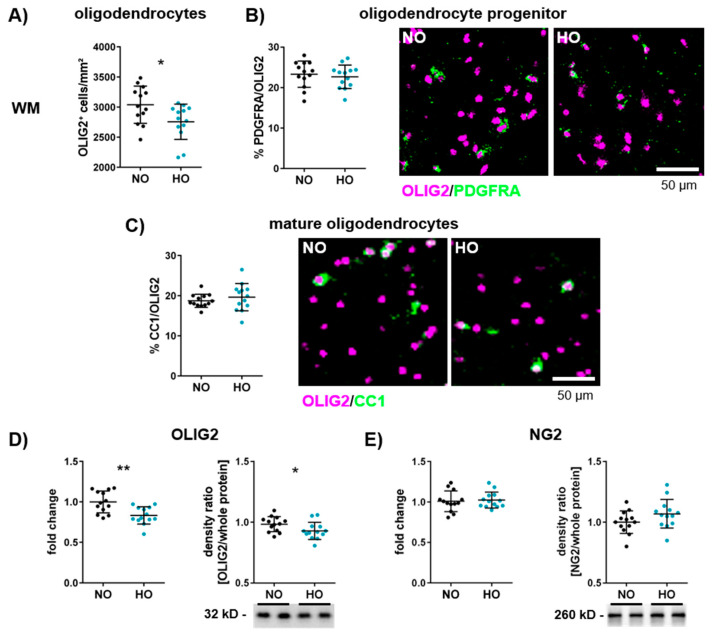
Hyperoxia reduces the number of oligodendrocytes in the developing white matter. On postnatal day 2 (P2), neonatal Wistar rats were exposed to 80% or 21% oxygen for seven days, and brains were investigated at P11. The absolute number of all oligodendrocytes (OLIG2-positive cells; (**A**)), the percentage of oligodendrocyte progenitor cells (PDGFRA/OLIG2; (**B**)) and mature oligodendrocytes (CC1/OLIG2; (**C**)) were quantified in the white matter (WM) at the hippocampal level (scale bar 50 µm). Gene and protein expression was analysed for the pan-oligodendrocyte marker OLIG2 (**D**) and the oligodendrocyte progenitor cell marker NG2 (**E**). Proteins of interest were normalised to the total protein, and reference blots are shown in the supplement (Figure S2). Data are presented in scatter plots with mean ± standard deviation; *n* = 13 animals/group; Student’s *t*-test: * *p* < 0.05, ** *p* < 0.01.

Oligodendrocytes are responsible for myelin synthesis and formation in the central nervous system (CNS) [50]. To assess whether the observed myelination deficit was associated with a reduced number of oligodendrocytes, brain tissue sections were stained with the pan-oligodendrocyte marker OLIG2. Animals exposed to hyperoxia showed a decrease in oligodendrocytes in the white matter and the thalamus (Figure 2A–C and Figure S3A–C). To determine the impact on oligodendrocyte maturation, OLIG2 staining was combined with staining of PDGFRA or CC1, markers for oligodendrocyte progenitor cells and mature oligodendrocytes, respectively (Figure 2B,C and Figure S3B,C). These analyses revealed no difference in the percentage of progenitor and mature cells in the white matter (Figure 2B,C), cortex and thalamus (Figure S3B,C). The reduction of oligodendrocytes was confirmed via mRNA and protein expression analyses (Figure 2D). Furthermore, gene and protein expression of NG2, an additional marker of oligodendrocyte precursor cells, were also not modulated (Figure 2E).

### 3.3. Altered Microglia Activation After One Week of Hyperoxia in the White Matter

**Figure 3:**
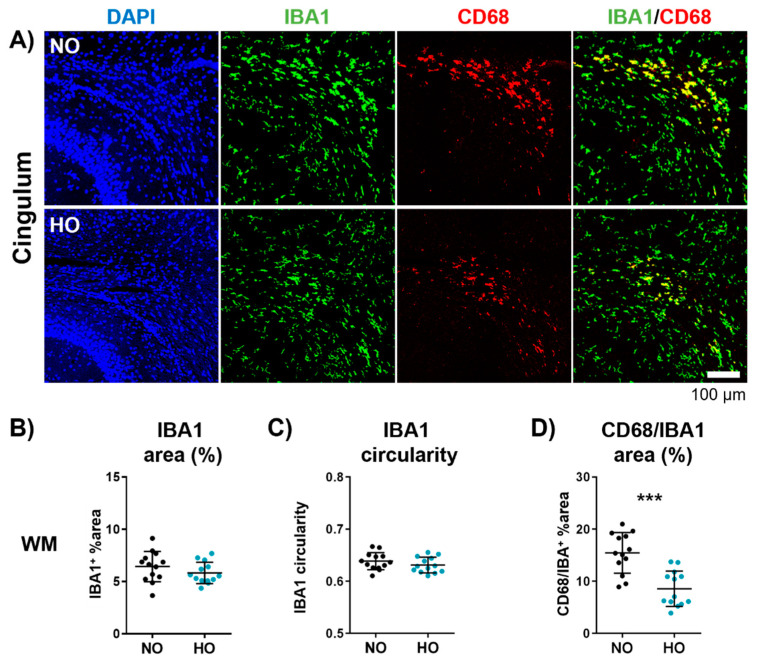
Hyperoxia reduces microglia activation in the white matter. Brains from animals exposed to hyperoxia or normoxia were collected at P11. Immunohistochemical double staining of IBA1/CD68 was performed to assess microglia cell density and activation in the white matter (WM). Exemplary images are shown for the cingulum of normoxia- and hyperoxia-treated animals ((**A**); scale bar 100 µm). The percentage of the IBA1-positive area (**B**), the cellular circularity of IBA1-positive cells (**C**), and the percentage of CD68-positive area from the total IBA1-positive area (**D**) were measured. Data are presented as scatter plots with mean ± standard deviation; *n* = 13 animals/group; Student’s *t*-test: *** *p* < 0.001; scale bar 1 mm.

As resident immune cells of the CNS, microglia have diverse and complex functions besides tissue development, homeostasis, and response to infection. They also interact with oligodendrocytes and have a direct impact on developmental myelination [51]. Hence, microglia activation was investigated via immunohistochemistry for IBA1 and CD68, specifically focusing on the white matter (cingulum, deep cortical, external capsule, and internal capsule) of the hippocampal level (Figure 3, Figures S4 and S5). While we detected no differences in IBA1 immunoreactivity, cellular circularity (Figure 3A–C, Figures S4A–C and S5), and protein expression (Figure S6A), we observed a pronounced reduction in CD68 expression on IBA1^+^ cells in hyperoxia-treated animals (Figure 3D, Figures S4D and S5). To analyse potential effects on cell polarisation, we assessed mRNA expression of the pro-inflammatory markers interleukin 12 (Il12) and Cd86, and the anti-inflammatory markers Arginase and Cd206. There was no difference between the normoxia and hyperoxia group (Figure S6B).

### 3.4. Postnatal Hyperoxia Leads to Hypervascularisation in the Immature Brain

**Figure 4:**
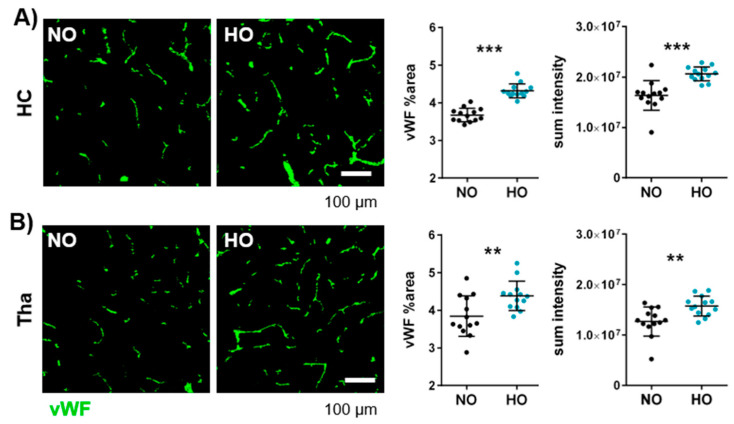
One week of hyperoxia induces hypervascularisation in the immature brain. After hyperoxia for seven days, vascularisation at the hippocampal level of eleven-day-old Wistar rats was investigated with immunohistochemistry for vWF. The percentage of vWF-positive area and sum pixel intensity of the staining were analysed in the hippocampus (**A**) and the thalamus (**B**). Data are presented in scatter plots with mean ± standard deviation; scale bar in (**A**,**B**) 100 µm; *n* = 13 animals/group; Student’s *t*-test (B: vWF %area) and Mann–Whitney U test (all other analyses): ** *p* < 0.01, *** *p* < 0.001.

In contrast to the well-known hypovascularisation in BPD lungs [52], far less is known about the effect of hyperoxia on brain vasculature. Hippocampal brain sections were stained against the vessel marker von Willebrand factor (vWF) and were analysed in the hippocampus, white matter, cortex, and thalamus (Figure S1). Interestingly, a significant increase in vWF-positive area and sum pixel intensity was detected in the hippocampus and thalamus (Figure 4) of hyperoxia-treated animals, while no effect was observed in cortex and white matter (Figure S7A,B). To check whether the increased vascular density is associated with an increased *Vegfa* expression, mRNA of *Vegfa* was analysed, revealing no difference between hyperoxia- and normoxia-treated animals (Figure S7C).

### 3.5. Neonatal Hyperoxia Causes an Arrest of Alveolarisation and Increases the Number of Macrophages in the Neonatal Lung

**Figure 5:**
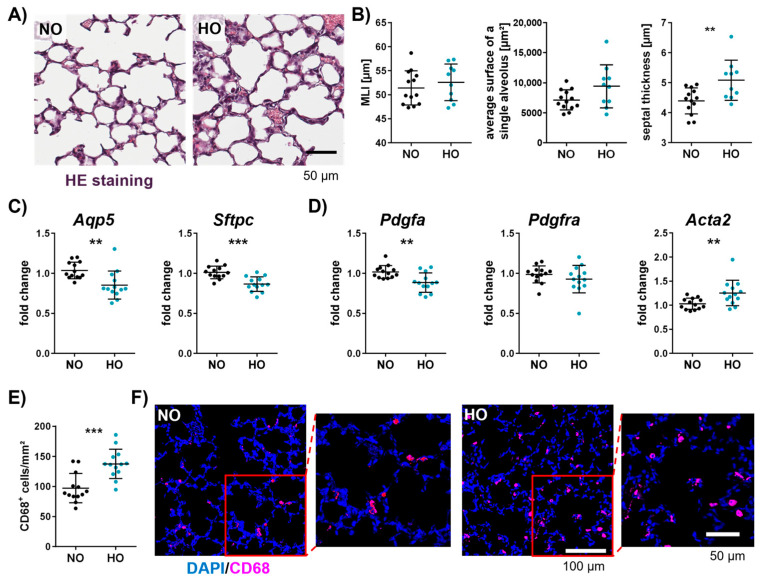
Neonatal hyperoxia increases septal thickness, number of macrophages, and altered gene expression of alveolar epithelial and mesenchymal cells in neonatal lungs. After hyperoxic or normoxic exposure from P2 to P9, lungs of eleven-day-old Wistar rat pups were collected and stained with haematoxylin and eosin (HE; (**A**); scale bar 50 µm) for quantitative histomorphometric analyses such as mean linear intercept (MLI), average surface of a single alveolus, and septal thickness (**B**). mRNA quantification of the right lung lobe was performed to analyse markers of alveolar epithelial cells (*Aqp5* and *Sftpc*; (**C**)) and mesenchymal cells (*Pdgfa*, *Pdgfra*, and *Acta2*; (**D**)). To investigate the number of macrophages, immunofluorescent staining was performed with the macrophage activation marker CD68 ((**E**,**F**); scale bar 100 µm, magnification scale bar 50 µm). Data are presented in scatter plots with mean ± standard deviation; NO group: *n* = 13 animals, HO group: *n* = 10–13 animals; Mann–Whitney U test (C: *Acta2* and *PDGFa* fold change) and Student’s *t*-test (all other analyses): ** *p* < 0.01, *** *p* < 0.001.

Besides deteriorating effects on brain myelination, altered microglia activation, and vascularisation, the effect of a seven-day hyperoxia exposure on lung morphology was investigated at P11. To examine the average free distance between gas exchange surfaces within the acinar airway complex [53], the mean linear intercept (MLI) was measured. While no significant differences were observed for the MLI, impaired alveolar development was indicated by an enlarged average surface of a single alveolus and a significant increase in septal thickness (Figure 5A,B). To further analyse the impact of hyperoxia on the alveolar epithelium, gene expression analysis of aquaporin 5 (*Aqp5*), a marker for alveolar epithelial cell type I (AEC I) and surfactant protein C (*Sftpc*), a marker for AEC II, were performed. Both were significantly reduced in lungs of hyperoxia-exposed animals (Figure 5C). Furthermore, platelet-derived growth factor A (*Pdgfa*), an important factor for alveolar septation, but not its receptor (*Pdgfra*) was decreased, whereas the myofibrotic marker actin alpha 2 (*Acta2*) was increased (Figure 5D). Interestingly, disturbed alveolarisation was associated with an increase in macrophages by approximately 40% in hyperoxia-treated animals (Figure 5E,F).

### 3.6. Neonatal Hyperoxia Induces Vascular Remodelling of Small Pulmonary Vessels

**Figure 6:**
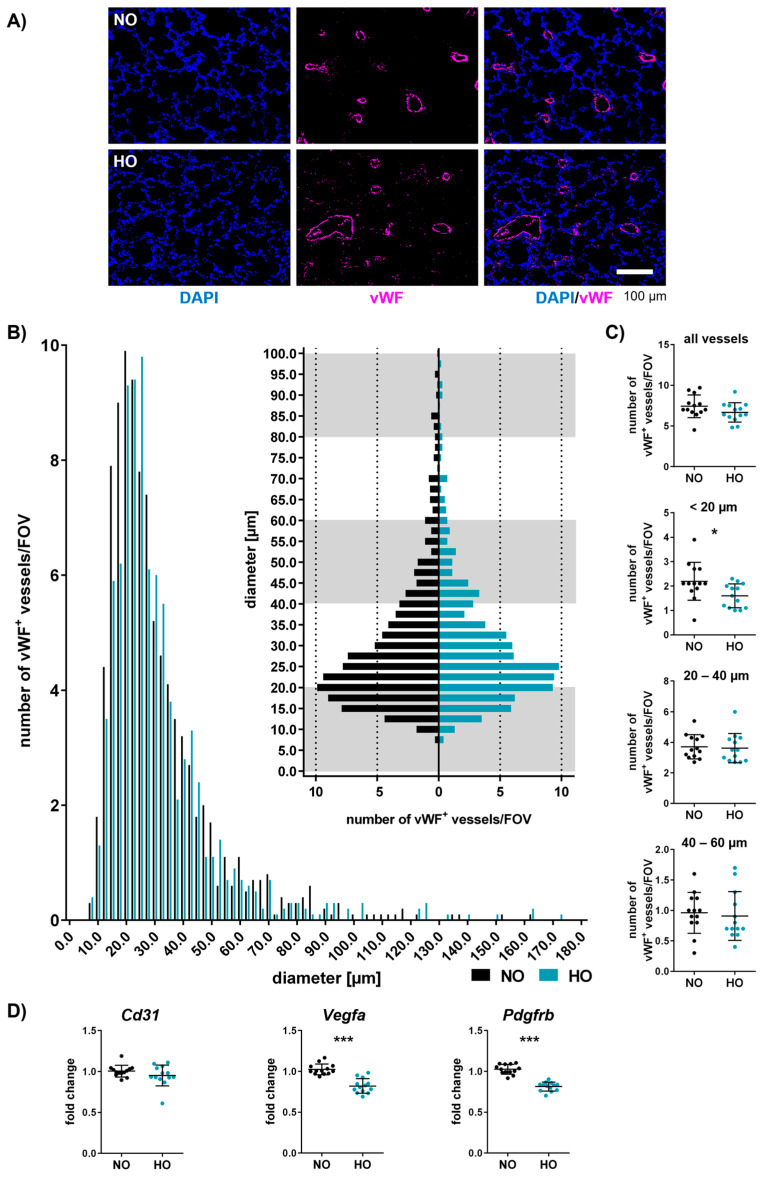
One week of neonatal hyperoxia reduces the number of pulmonary microvessels. Neonatal rats were exposed to 80% or 21% oxygen from P2 to P9. Lung vascularisation was analysed at P11 with immunohistochemistry staining using vWF, and large-scale images of the whole lung section were taken. The diameter and number of the vWF^+^ vessels (**A**) were measured in ten random, non-overlapping fields. Distribution of the vessel diameter [µm] versus the number of vessels per field of view (FOV) (**B**) and vessel density per FOV grouped into all vessels, smaller than 20 µm, 20–40 µm, and 40–60 µm were evaluated (**C**). mRNA quantification of the right lung lobe was performed to analyse markers involved in vascularisation (*Cd31*, *Vegfa*, and *Pdgfrb*; (**D**)). Data are presented in scatter plots with mean ± standard deviation; *n* = 13 animals/group; Mann–Whitney U test ((**C**): number of vWF^+^ vessels between 20 and 40 µm; (**D**): *Cd31* fold change) and Student’s *t*-test (all other analyses): * *p* < 0.05, *** *p* < 0.001. Histogram visualises vessel number versus vessel size distribution.

Impaired alveolarisation is accompanied by an altered vascular architecture, typically detected in BPD models [52]. Vessel diameter and density were investigated at P11 in hyperoxia-treated and control animals via immunohistochemistry against vWF (Figure 6A). The distribution of vessel number/FOV to vessel diameter was visualised in histograms (range from 0 to 180 µm and an enlarged histogram version from 0 to 100 µm; Figure 6B). While the total number of vessels (up to 180 µm) was not altered (Figure 6C), we detected a prominent decrease in the number of small vessels smaller than 20 µm (Figure 6B,C). Gene expression analysis revealed that the vessel marker CD31 was not altered, whereas *Vegfa*, important for vasculogenesis and angiogenesis, was significantly reduced in lungs of animals exposed to hyperoxia (Figure 6D). Additionally, we observed a reduction in the pericyte marker *Pdgfrb* (Figure 6D), which is important for vascular stability and growth [54].

### 3.7. Myelination, Microglia Activation, and Vascularisation in the Brain Correlate with Lung Vascularisation and Gene Expression of Alveolar Markers

**Figure 7:**
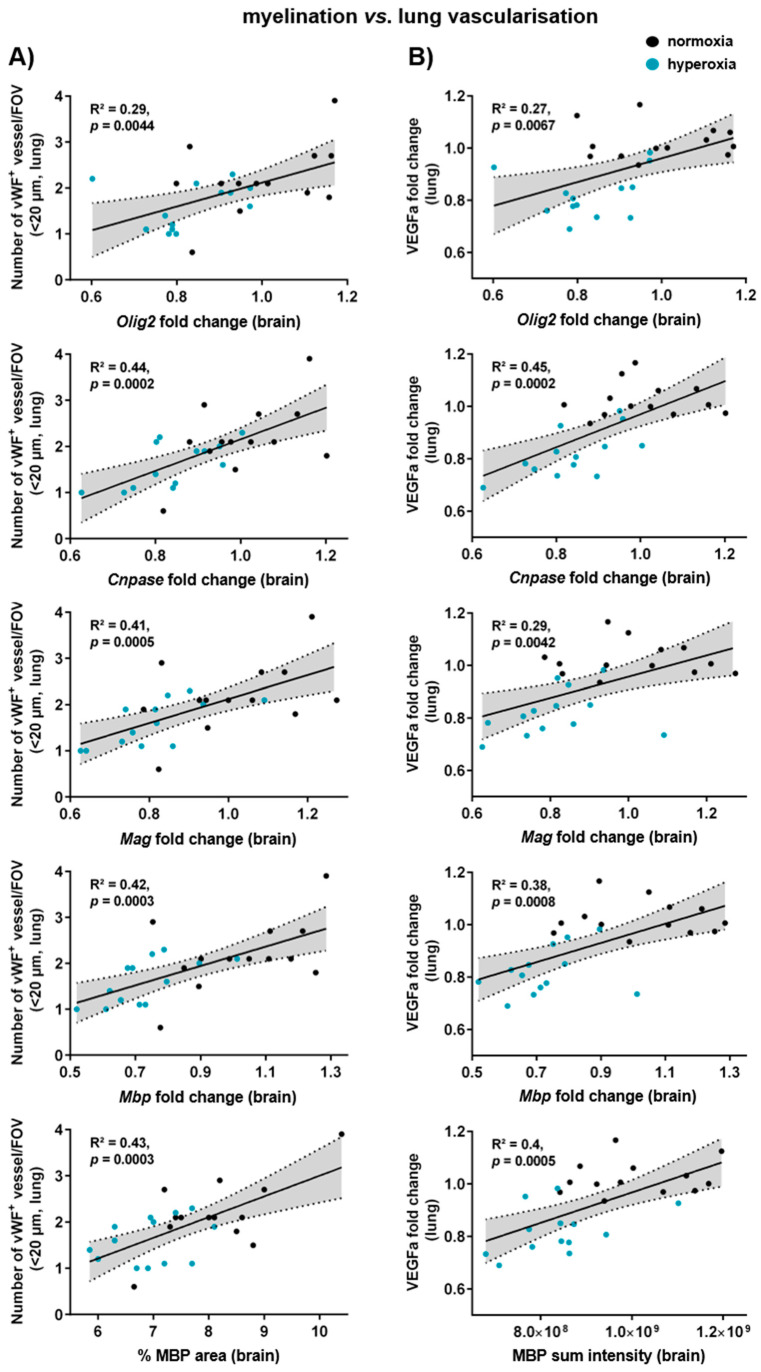
Myelination in the brain positively correlates with micro-vascularisation in the lung. Neonatal rats were exposed to 21% or 80% oxygen from P2 to P9 followed by real-time PCR and immunohistochemistry analyses of the brain and the lung at P11. Gene and protein expression of myelin-associated proteins (*Olig2*, *Cnpase*, *Mag*, and *Mbp*) were correlated with the number of microvessels (**A**) and gene expression levels of the angiogenesis factor *Vegfa* (**B**) in the lung. Correlation analyses are presented with a 95% confidence interval (grey area) togetherwith the coefficient (R^2^) and corresponding *p* value. *X*-axes: brain values; *Y*-axes: lung values; black points: normoxia group; blue points: hyperoxia group.

Though hyperoxia is a major risk factor for BPD and EoP, direct correlations between both affected organs are missing. To investigate a potential lung–brain axis, we performed correlation analysis of brain and lung injury parameters in our model. Indeed, we detected positive correlations and clear group differences between the number of microvessels (<20 µm) in the lung and gene expression levels of *Olig2*; the myelin-associated proteins *Cnpase*, *Mag*, and *Mbp*; and the MBP-positive area in the brain (Figure 7A). Similarly, a positive correlation was determined between myelin parameters and *Vegfa* gene expression in the lung (Figure 7B). Interestingly, markers for alveolar epithelium and mesenchymal cells (*Sftpc*, *Aqp5,* and *Pdgfrb*) in the lung were also positively correlated with myelination (Figure S8A) and microglia activation (Figure S8B) in the brain. Furthermore, hippocampal vascularisation was positively correlated with the number of macrophages in the lung and negatively correlated with lung *Vegfa* expression, as well as markers of alveolarisation and septation (Figure S8C).

## 4. Discussion

Despite improvements in neonatal intensive care of very preterm infants, long-term consequences such as BPD and EoP remain a major problem, leading to lifelong health, social and economic burden for infants and their families [55]. Because of the paucity of studies focusing on both organs in one experimental model, it is still unclear why infants suffering from BPD are at higher risk for adverse neurodevelopmental outcome. White matter injury is one of the most common hallmarks of EoP and associated neurodevelopmental deficits [56]. In the present work, we show that one week of hyperoxia with 80% oxygen initiated at P2 leads to pronounced hypomyelination, supported by reduced gene and protein expression of important myelin proteins, including MBP, MAG, and CNPase at term-equivalent age, i.e., two days after hyperoxia. Our results confirm previous studies on EoP, which demonstrated hypomyelination immediately following hyperoxia exposure [31,57]. Hyperoxia-induced myelination deficit was previously associated with long-term alterations in myelin structures and motor–cognitive impairment persisting into adulthood [31,32,58]. Whether the present model with seven days of hyperoxia has a long-lasting impact on myelin formation and neurodevelopmental outcome will be investigated in future studies.

The frequently observed hypomyelination is attributed to an increased susceptibility of immature oligodendrocytes, as shown in EoP models with short-term hyperoxia of 24–48 h at P6, resulting in disturbed oligodendrocyte proliferation and maturation, and increased degeneration [31,59]. In line with these findings, Pham et al. detected a similar impact of a longer duration of hyperoxia. As such, 80% oxygen applied from birth to P7 resulted in a decrease of immature oligodendrocytes during hyperoxia at P3, followed by delayed maturation of oligodendrocytes at P10 [44]. Accordingly, the observed myelination deficits in the present work were associated with an overall reduction of oligodendrocytes. The percentage of oligodendrocyte progenitor cells and mature oligodendrocytes did not differ, suggesting that seven days of hyperoxia affect oligodendrocytes at different developmental stages, including immature, mature myelinating or not yet myelinating oligodendrocytes.

Oligodendrocyte maturation and myelination are influenced by a variety of factors and cells, with microglia playing a prominent role [51]. For instance, microglia in the white matter exhibit a pronounced expression of genes driving oligodendrocyte maturation and myelination compared to microglia cells in the cortex [60]. Furthermore, microglia are suggested to regulate myelin growth and correct formation [61]. We previously demonstrated an increased microglia activation after 24 h of hyperoxia (80% O_2_) at P7, revealed by an increased expression of IBA1 and a more amoeboid shape [31]. Interestingly, in the present study, with seven days of 80% oxygen between P2 and P9, we did not observe alterations in IBA1 immunoreactivity and morphology at P11. Despite unchanged IBA1, microglia activation, identified by CD68 staining, was reduced in the hyperoxia group. Reduced CD68 expression seems to contradict previous work, suggesting that myelination deficits but also EoP are associated with increased microglia activation [44,62]. However, it has to be considered that CD68 is indicative for phagocytosis and microglia play an important role in physiological brain development for example through synaptic pruning as well as phagocytosing oligodendrocyte precursor cells and myelin [63]. Given the present results with reduced CD68 expression, impaired myelination might be related to impaired microglia function. Furthermore, CD68 is only one single marker, which might not solely characterise activated and pro-inflammatory microglia [64]. This is supported by a previous study in a model of adult stroke, which revealed increased transforming growth factor beta expression, specifically in CD68^+^ microglia [65]. Though we did not detect differences in cell polarisation in the hyperoxia group at P11, this might be explained by differences in tissue sampling, i.e., we used brain lysates of the hippocampal level instead of isolated microglia. Therefore, further analysis is needed to investigate the impact of hyperoxia on microglia polarisation. Together, these results suggest a distinct temporal regulation of microglia responses depending on the time of onset and duration of experimental hyperoxia and on the time of sample assessment following the insult.

Besides hypomyelination and altered microglia activity, we analysed the effect of hyperoxia on the brain vasculature. Even though the capillary network is still developing in the early postnatal phase of rodents, the potential impact of oxygen excess is not well understood [66]. In the present work, hyperoxia-exposed animals revealed hypervascularisation in the hippocampus and thalamus at P11, which seems to be in contrast to two BPD studies, showing a decreased cortical vascular network that persists into adulthood [43,67]. Differences in brain regions (cortex vs. hippocampus/thalamus), varying time points in the initiation and/or durations of hyperoxia (14 days at P0 vs. 7 days at P2), and lastly, different analysis time points (P6/P14/P30 vs. P11) may account for these discrepancies. However, the effect of an increased vessel density might be transient, as previously reported in a model of intermitted hyperoxia–hypoxia, where an increased cortical vascular network was noticed at P14 immediately after hyperoxia that normalised to control levels at P28 [68]. Additionally, long-term consequences of this transient hypervascularisation for brain development and function remain unclear. From studies in models of retinopathy of prematurity (ROP), it is known that hyperoxia induces excessive vessel proliferation and formation, which can result in a disturbed neurovascular unit, fibrous tissue, and retinal detachment [69,70]. Further analyses are required to investigate whether the vascular remodelling in the hippocampus and thalamus in our model persists, or whether newly formed vessels regress over time and which effect the early hyperoxia-induced hypervascularisation may have on neurovascular unit development and long-term neurodevelopmental outcome.

In contrast to the brain, a reduced pulmonary vascular density was shown in different models of BPD [44,52,71,72]. In line with that, the present analysis following one week of hyperoxia revealed a diminished number of small vessels, especially those smaller than 20 µm, suggesting impaired angiogenesis. This is further supported by reduced *Vegfa* levels in hyperoxia-treated animals. VEGFa is an important factor in angiogenesis, and experimental VEGFa gene therapy revealed an increased vessel density in hyperoxia-treated animals [73]. In addition to impaired angiogenesis, hyperoxia may disturb the formation and/or maturation of vessels, as Alphonse and colleagues demonstrated that lung endothelial colony-forming cells form less cordlike endothelial networks after hyperoxic exposure [74]. This might be a plausible explanation for the microvessel reduction in hyperoxia-treated animals demonstrated in this study.

Proper vascularisation is closely related to alveolarisation during lung development [75,76]. This is supported by the fact that PDGFRB is involved in endothelial and epithelial development of the lung and that reduced PDGFRB levels were associated with impaired alveolarisation [77,78]. Furthermore, PDGFa-deficient mice showed alveolar arrest and reduced VEGFa expression [78,79]. Interestingly, the detrimental effects of one week of hyperoxia on the small vessel density observed in the present work were associated with pronounced reductions in all three genes, i.e., *Vegfa*, *Pdgfa*, and *Pdgfrb*, which might explain the vascular alterations. Furthermore, aquaporin 5 (AQP5) and surfactant protein C (SFTPC), markers for alveolar epithelial cell (AEC) type I and type II, were reduced in hyperoxia-treated animals. These findings are in line with a reduced SFTPC expression, as well as an increased AEC II depletion after hyperoxia in a mouse model of BPD after 28 days of hyperoxia with 85% oxygen [46]. Of note, molecular alterations detected in the present work were associated with a moderate level of disrupted alveolarisation, revealed by an increased average surface of a single alveolus and thickened septal walls, as previously noticed in models of BPD with longer hyperoxia durations [46,80]. Our results highlight the high vulnerability of the immature lung even to short durations of high oxygen levels. Interestingly, morphological changes were accompanied by a strong increase in infiltrated macrophages, which is in line with previous work [46,81]. While alveolar resident macrophages are indispensable for the normal alveolarisation process, hyperoxia-mediated activation of alveolar macrophages leads to the recruitment of peripheral macrophages, excessive cytokines release, and epithelial cell damage [81,82]. This finally leads to a BPD-like phenotype, characterised by the failed formation of alveoli and vessels. 

In our hands, one week of hyperoxia simultaneously induced brain and lung injury with a distinct correlation between pulmonary microvasculature and brain myelination. Given the close relation between vascularisation and alveolarisation in the lung, it does not seem surprising that poor myelination is also correlated with a diminished expression of alveolar and septal markers. In line with that, Kim et al. demonstrated that impaired alveolarisation correlates with disrupted myelination, indicated by an increased MLI in the lung and a reduced MBP intensity in the brain of hyperoxia-treated animals [41]. Still, the vast majority of published studies did not perform correlation analysis of lung and brain injury, thereby hampering direct conclusions on the lung–brain axis. One hypothesis suggests that the lung and brain interact through inflammatory processes. For instance, extracellular vesicles (EVs) extracted from the lungs of hyperoxia-exposed animals, enriched with pro-inflammatory cytokines, have been shown to disrupt alveolarisation in the lung. Further analysis revealed that EVs cross the blood–brain barrier and activate microglia in naïve rats, thereby contributing to injuries resembling EoP and BPD [39]. Furthermore, a major task in future work will be the identification of the cellular and molecular links connecting both organ injuries.

Here, we provide a novel experimental model in neonatal rats showing that a relatively short and moderate exposure of seven days of hyperoxia with 80% oxygen induces pronounced deficits in lung and brain development, characterised by hypovascularisation and impaired alveolarisation in the lung. Importantly, changes in the lung correlated with pronounced myelination deficits, hypervascularisation, and unexpected microglial changes in the brain. The present study focused on the investigation of oxygen toxicity in immature organs, acknowledging the significant influence of a major noxious stimulus, i.e., hyperoxia, which can hardly be avoided due to physiological differences between in utero and ex utero conditions [9]. This is further exacerbated by clinical interventions (e.g., mechanical ventilation) often needed to ensure survival of preterm infants [14,15]. Despite advances in experimental research, therapeutic interventions for the treatment of EoP and BPD are limited [83,84]. With the present experimental model, we provide a novel tool not only to investigate mechanisms potentially linking both organ injuries but also to evaluate novel therapeutic approaches, which, in the best case, treat both severe preterm-birth-related complications. First attempts, including, for example, stem cell-based therapies, have been made in typical BPD models with longer hyperoxia exposures [40,41,42,44]. However, varying outcomes were reported in both organs, highlighting the urgent need for future studies to improve our understanding of the pathophysiological link between brain and lung injury in the context of oxygen toxicity.

## Data Availability

The original data shown in this manuscript are available from the corresponding author upon reasonable request.

## Supplementary Materials

The following supporting information can be downloaded at: https://www.mdpi.com/article/10.3390/cells14060443/s1, Figure S1: Schematic illustration of brain regions analysed via immunohistochemistry; Figure S2: Original full-length western blots; Figure S3: Seven days of hyperoxia lead to a reduction in oligodendrocytes in the thalamus but not in the cortex; Figure S4: Reduced microglia activation in different white matter regions after seven days of hyperoxia; Figure S5: Exemplary images of microglia activation in different white matter regions after seven days of hyperoxia; Figure S6: Neonatal hyperoxia did not alter IBA1 protein expression or cell polarisation; Figure S7: Vascularisation in the cortex or white matter was not affected after one week of hyperoxia; Figure S8: Myelination, microglia activation, and vascularisation in the brain correlate with alveolarisation and septation markers in the lung; Table S1: Antibodies used for immunohistochemistry on brain and lung tissue sections; Table S2: Antibodies used for Western blot analysis of brain samples; Table S3: TaqMan Assays used for mRNA expression analysis in brain and lung tissues.

## Abbreviations

The following abbreviations are used in this manuscript:

ACTA2	Actin alpha 2
AQP5	Aquaporin 5
BSA	Bovine serum albumin
CA	Cornu ammonis
CC1	Adenomatous polyposis coli clone CC1
CD206	Cluster of differentiation 206
CD31	Cluster of differentiation 31 or platelet endothelial cell adhesion molecule 1
CD68	Cluster of differentiation 68 or macrosialin
CD86	Cluster of differentiation 86
Cing	Cingulum
CNPase	2′,3′-cyclic-nucleotide 3′-phosphodiesterase
CTX	Cortex
Dapi	4′,6-diamidino-2-phenylindol
DC	Deep cortical
DG	Dentate gyrus
EC	External capsule
FOV	Field of view
HC	Hippocampus
HE	Haematoxylin–eosin
HO	Hyperoxia
Iba1	Ionised calcium-binding adapter molecule 1
IL12	Interleukin 12
IntC	Internal capsule
MAG	Myelin-associated glycoprotein
MBP	Myelin basic protein
MLI	Mean linear intercept
MP	Non-fat milk powder
NG2	Neural/glial antigen 2
NO	Normoxia
Olig2	Oligodendrocyte transcription factor 2
P	Postnatal day
PBS	Phosphate-buffered saline
PDGFRA	Platelet-derived growth factor receptor alpha
PDGFRB	Platelet-derived growth factor receptor beta
PFA	Paraformaldehyde
PGDFa	Platelet-derived growth factor A
SFTPC	Pro-surfactant protein C
TBS	Tris-buffered saline
TBS-T	0.1% Tween-20 in TBS
Tha	Thalamus
VEGFa	Vascular endothelial growth factor A
vWF	von Willebrand factor
WM	White matter
