# Supplementary material for: A Novel Model for Simultaneous Evaluation of Hyperoxia-Mediated Brain and Lung Injury in Neonatal Rats
Source: Cells. 2025 Mar 16;14(6):443. doi: 10.3390/cells14060443 (PMC11941478; doi:10.3390/cells14060443)
Supplement: Supplementary file 1 [file cells-14-00443-s001.zip › cells-3500213-supplementary.pdf]

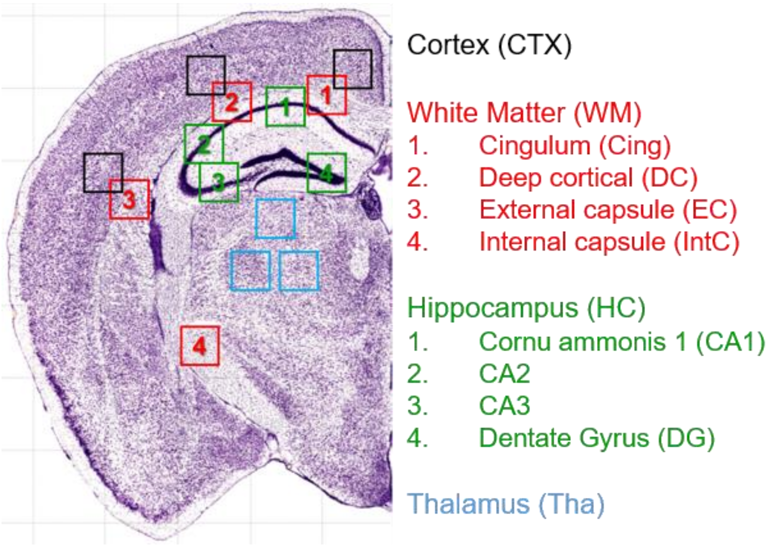

Figure S1: Schematic illustration of brain regions analysed via immunohistochemistry. Brains of neonatal pups were collected at P11. Immunohistochemistry was performed in hippocampal brain sections and large images were taken by confocal microscopy. Three to four field of views were analysed in the cortex (black squares), white matter (red squares), hippocampus (green squares) and thalamus (blue squares).

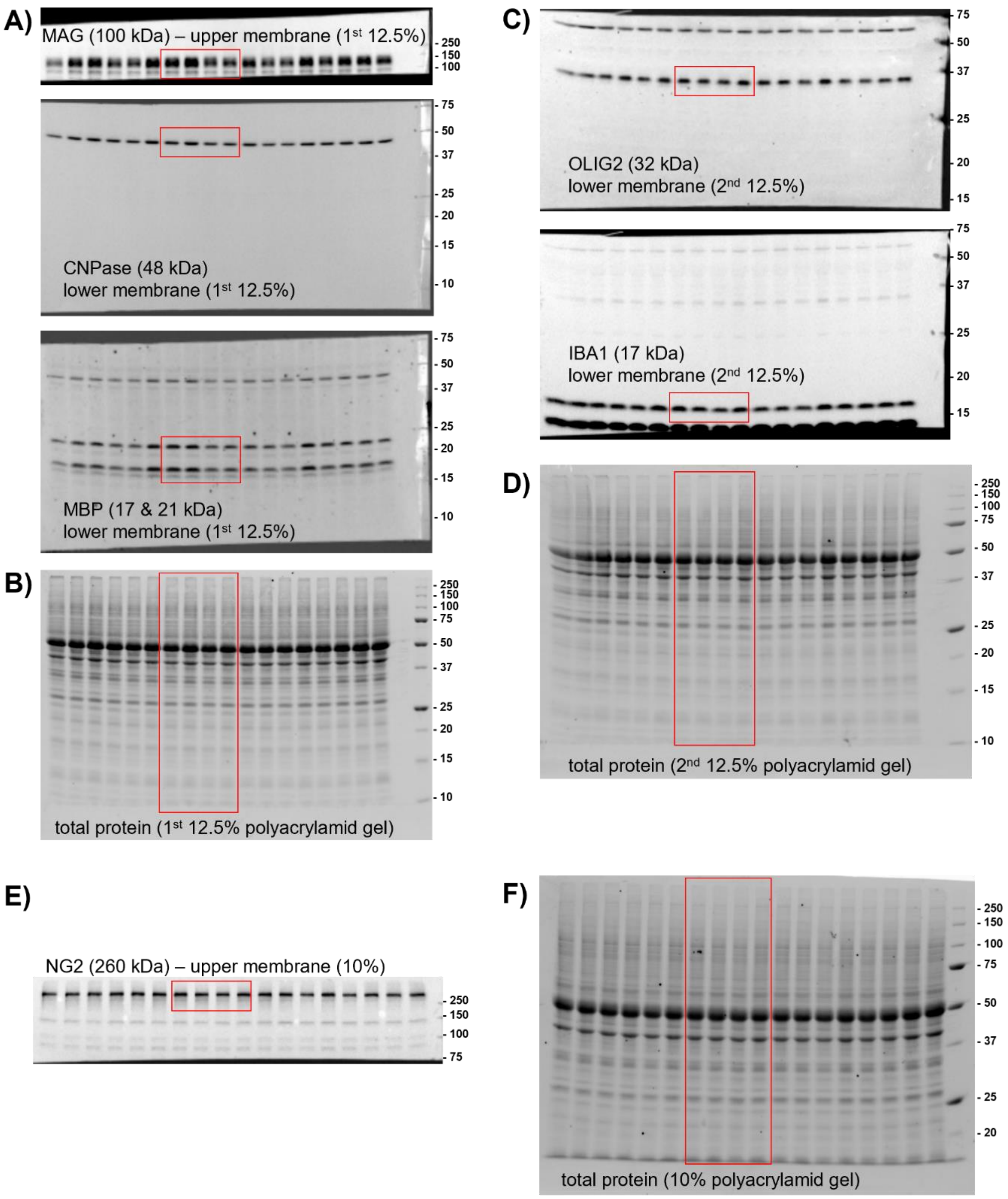

Figure S2: Original full-length western blots. All images represent merged images of original western blot images and transmitted light images to visualise ladder bands (unstained marker). To avoid cross reactions of primary or secondary antibodies membranes were cut at approximately 75 kDa prior to antibody incubation and detection. Analysis of MAG, CNPase and MBP (A) was performed on one membrane (1st 12.5% polyacrylamide gel; B). OLIG2 and IBA1 (C) were detected on another membrane (2nd 12.5% polyacrylamide gel; D). Due to protein size analysis of NG2 (E) was performed on an additional membrane (10% polyacrylamide gel; F). Protein expression was normalised to total protein content (B, D and F). Red boxes indicate cropped regions of proteins of interests used for illustrations in the main figures of the manuscript.

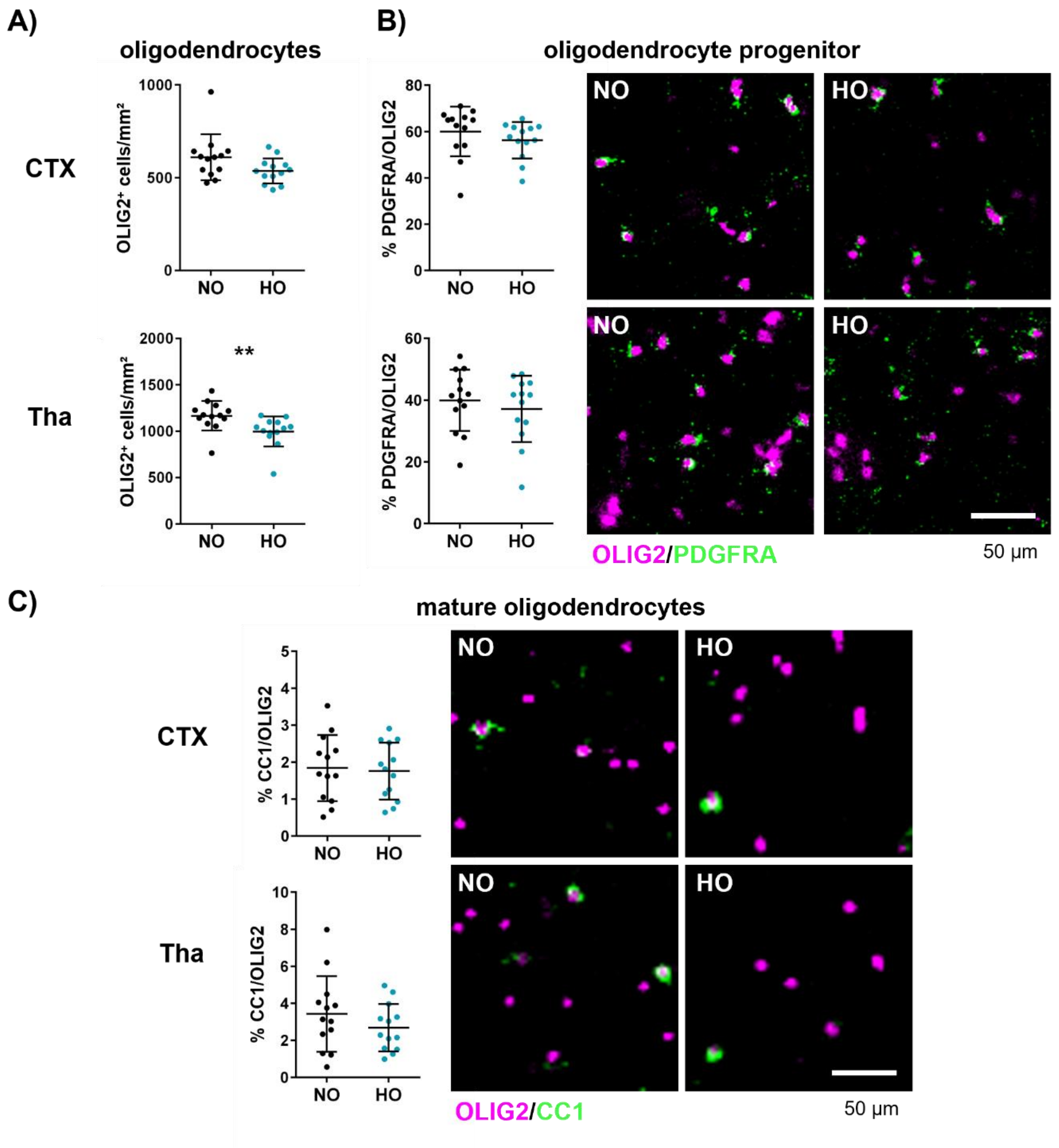

Figure S3: Seven days of hyperoxia lead to a reduction of oligodendrocytes in the thalamus but not in the cortex. On postnatal day 2 (P2), neonatal Wistar rats were exposed to 80% or 21% oxygen for 7 days and brains were investigated at P11. Total oligodendrocyte cell numbers (OLIG2; A), the percentage of oligodendrocyte progenitor cells (PFGFRA; B) and mature oligodendrocytes (CC1; C) were evaluated in the cortex and thalamus (scale bar 50 µm). Data are presented in scatter plots with mean ± standard deviation; n = 13 animals/group; Mann-Whitney U test (A and B: CTX - % PDGFRa/OLIG2) and Student's *t*-test (B: Tha – % PDGFRa/OLIG2 and C): *$p < 0.05$, **$p < 0.01$.

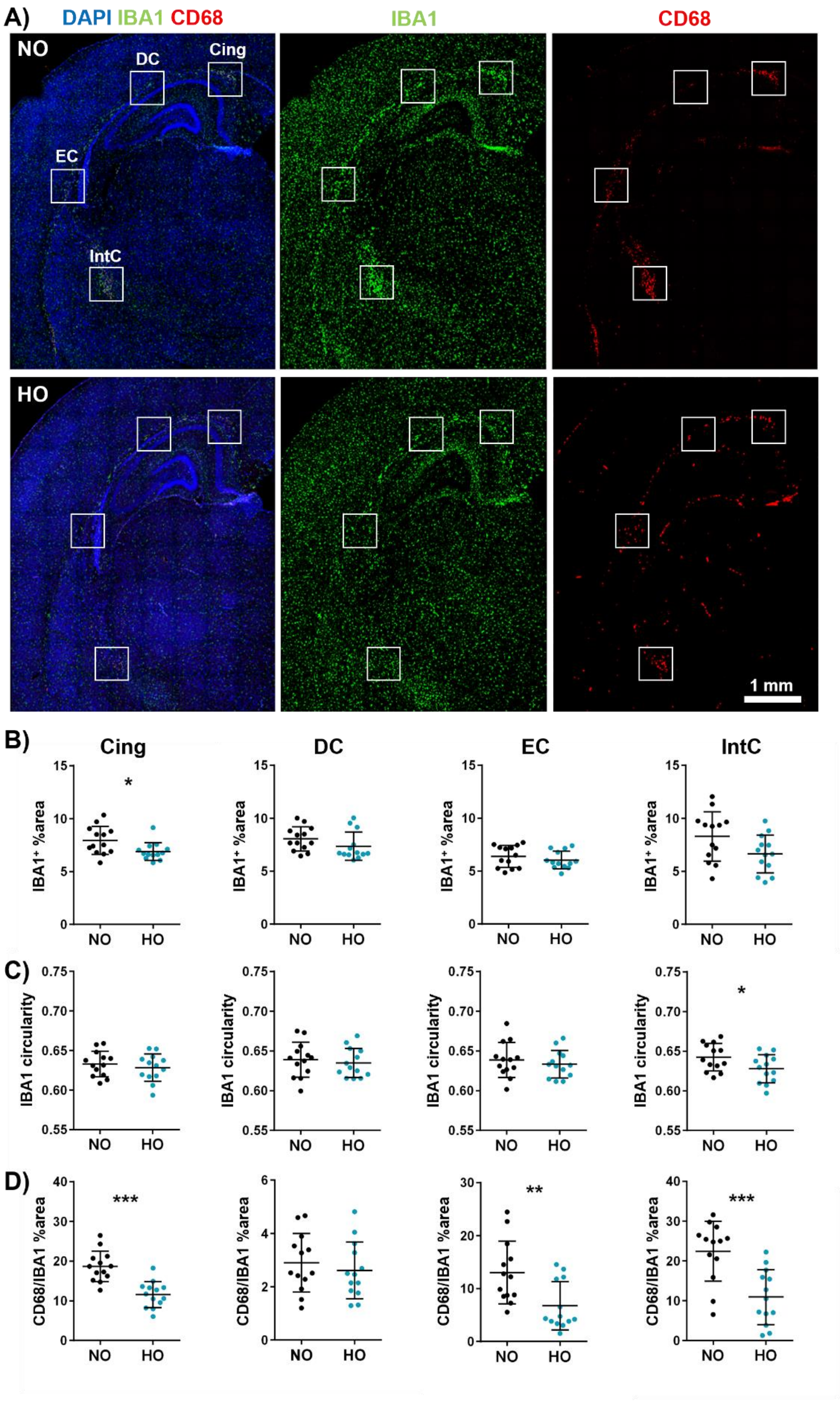

Figure S4: Reduced microglia activation in different white matter regions after seven days of hyperoxia. On postnatal day 2 (P2), neonatal Wistar rats were exposed to 80% or room-air (21% oxygen) for one week and remained under room-air for two more days. Brains were collected at P11 and double stained for IBA1 and CD68 (A). Analyses were performed in different white matter regions: cingulum (Cing), deep cortical white matter (DC), external capsule (EC) and internal capsule (IntC). The percentage of the IBA1 positive area (B) and circularity of IBA1 positive cells (C) and the percentage of the CD68 positive area from the IBA1 positive area (D) were quantified. Data are presented as scatter plots with mean ± standard deviation; n = 13 animals/group; Mann-Whitney U test (B: Cing - IBA1 %area) and Student's *t*-test (all other analyses): *$p < 0.05$, **$p < 0.01$, ***$p < 0.001$.

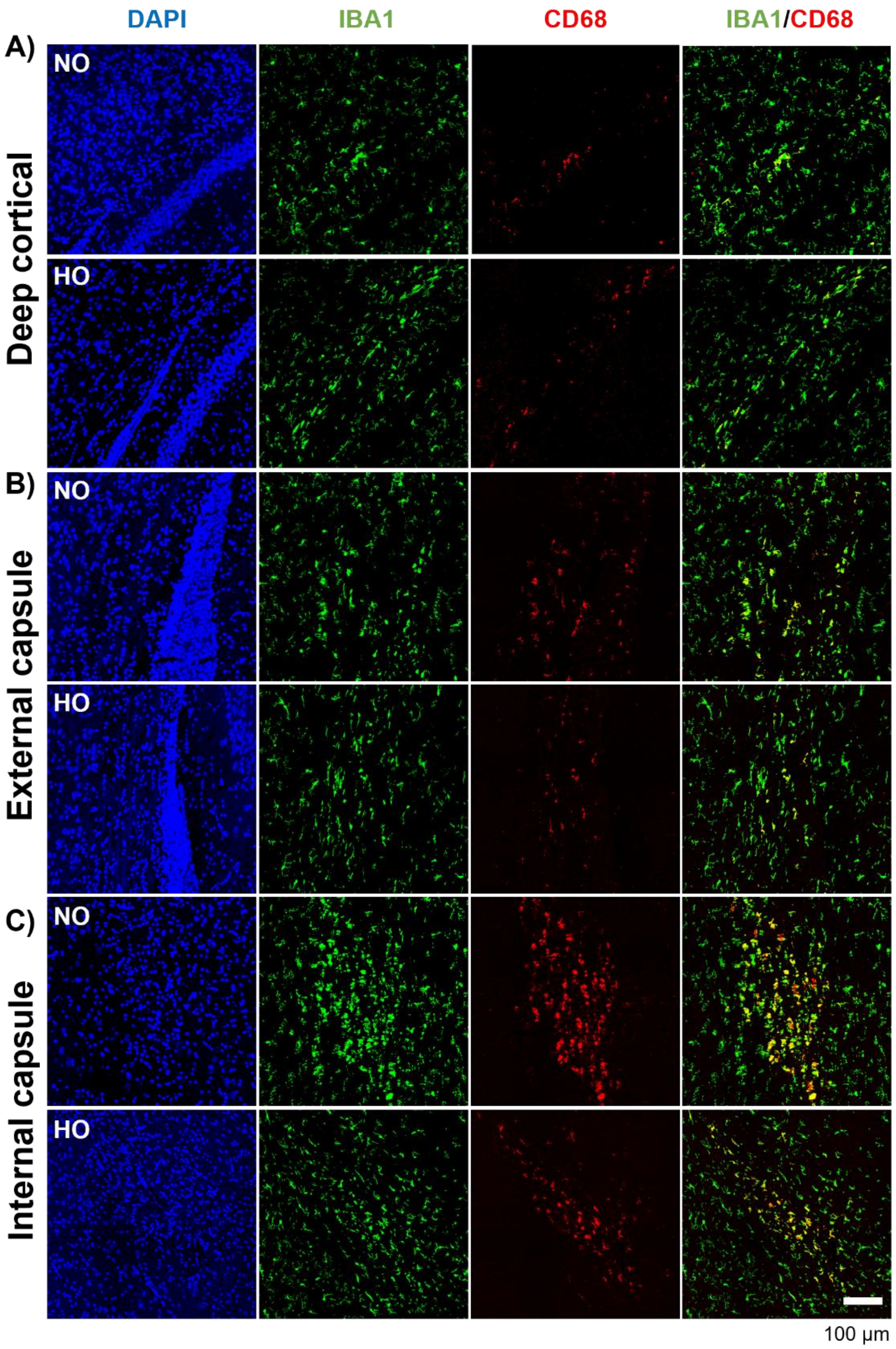

Figure S5: Exemplary images of microglia activation in different white matter regions after seven days of hyperoxia. On postnatal day 2 (P2), neonatal Wistar rats were exposed to 80% or room-air (21% oxygen) for one week and remained under room-air for two more days. Brains were collected at P11 and double stained for IBA1 and CD68. Exemplary images of the IBA1 and CD68 double staining are shown in the deep cortical white matter (A), external capsule (B) and internal capsule (C) for the normoxia and the hyperoxia group (scale bar 100 µm).

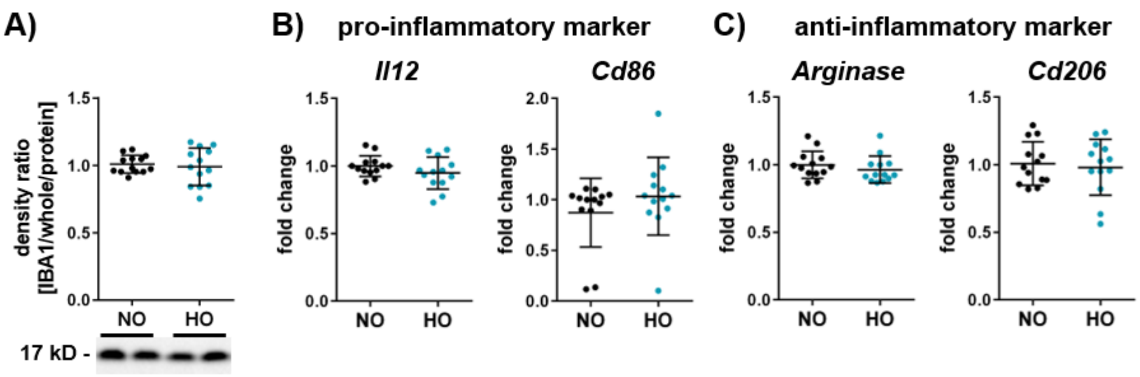

Figure S6: Neonatal hyperoxia did not alter IBA1 protein expression or cell polarisation. Brains of hyperoxia- or normoxia-exposed animals were removed at P11 and mRNA as well as protein were isolated from hippocampal sections. Protein expression analysis of IBA1 was performed via western blot (A). IBA1 were normalised to the total protein. The reference blot is shown in the supplement (Figure S2). Expression of pro-inflammatory (*Il12* and *Cd86*; B) and anti-inflammatory (*Arginase* and *Cd206*; C) markers were quantified via real time PCR. Data are presented as scatter plots with mean ± standard deviation; n = 13 animals/group.

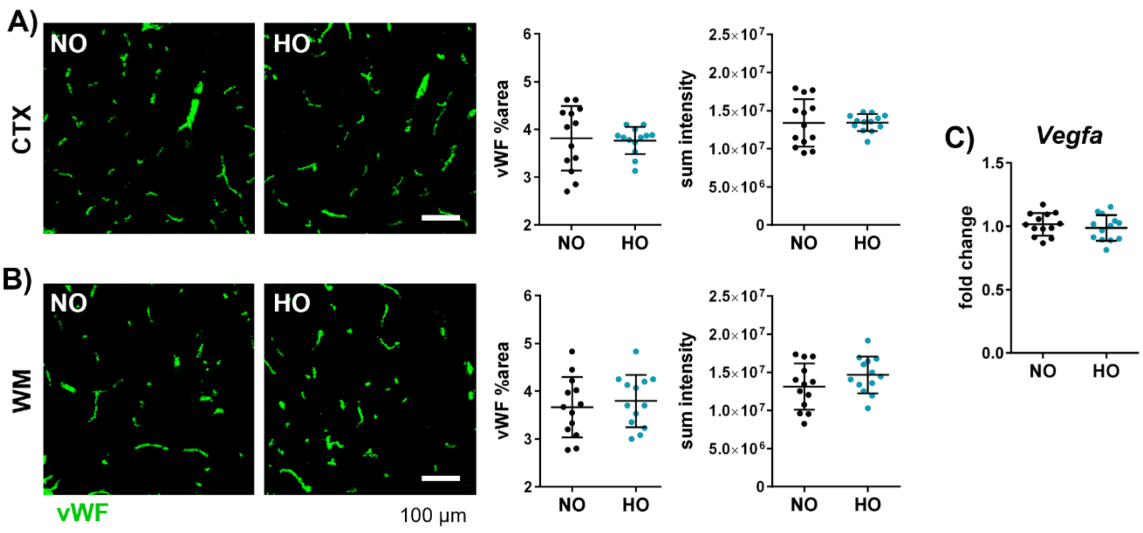

Figure S7: Vascularisation in the cortex or white matter was not affected after one week of hyperoxia. Vessel analysis was performed at the hippocampal level after one week of hyperoxia or normoxia at P11. The percentage of the vWF positive area and sum pixel intensity of three regions of interest were measured in the cortex (A) and the white matter (B). Gene expression of *Vegfa* was analysed with real time PCR in tissue lysates from the hippocampal level (C). Data are presented as scatter plots with mean ± standard deviation; n = 13 animals/group; Scale bar 100 µm.

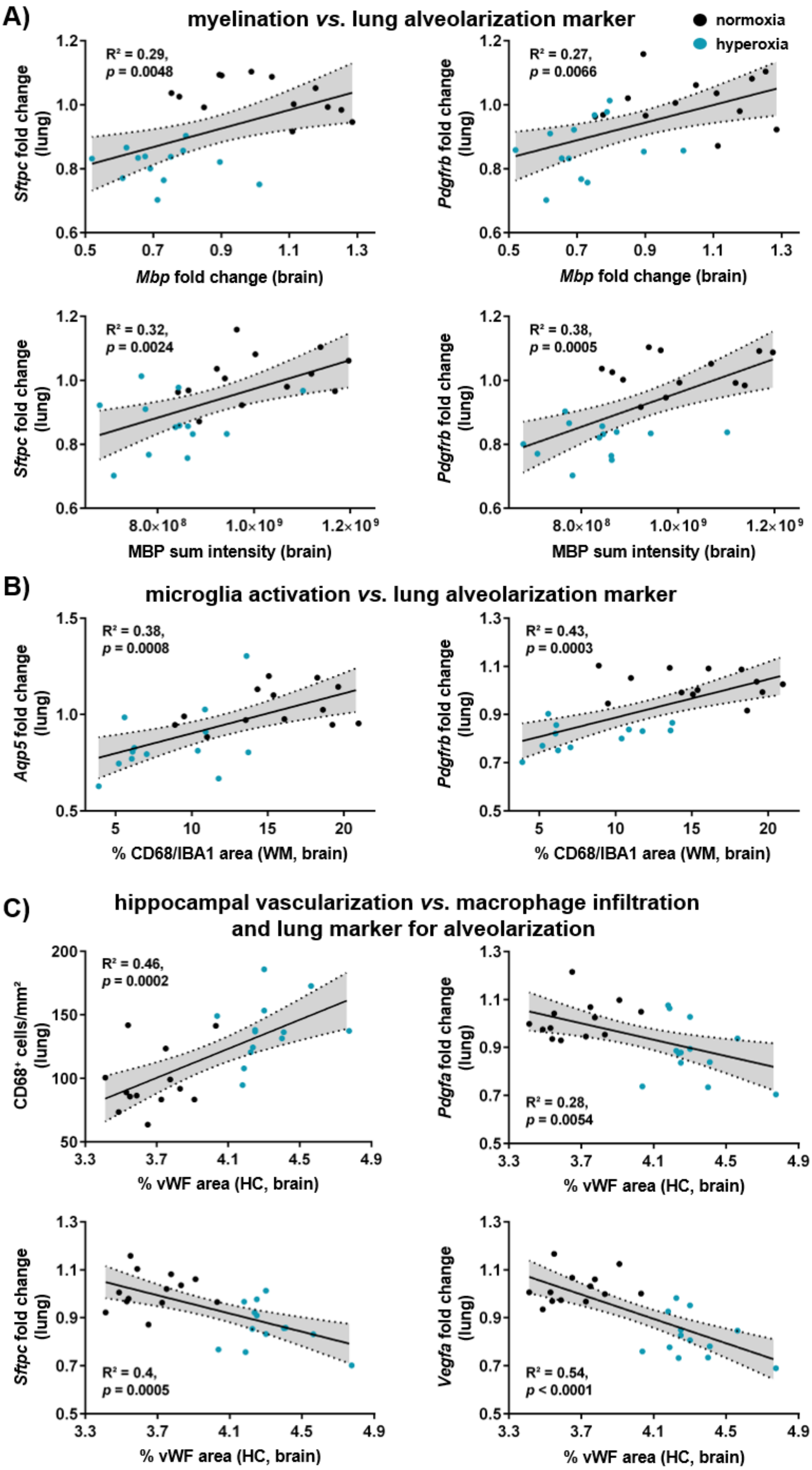
A)
myelination vs. lung alveolarization marker
normoxia
hyperoxia
R² = 0.29,
p = 0.0048
Sftpc fold change (lung)
Mbp fold change (brain)
R² = 0.27,
p = 0.0066
Pdgfrb fold change (lung)
Mbp fold change (brain)
R² = 0.32,
p = 0.0024
Sftpc fold change (lung)
MBP sum intensity (brain)
R² = 0.38,
p = 0.0005
Pdgfrb fold change (lung)
MBP sum intensity (brain)
B)
microglia activation vs. lung alveolarization marker
R² = 0.38,
p = 0.0008
Aqp5 fold change (lung)
% CD68/IBA1 area (WM, brain)
R² = 0.43,
p = 0.0003
Pdgfrb fold change (lung)
% CD68/IBA1 area (WM, brain)
C)
hippocampal vascularization vs. macrophage infiltration
and lung marker for alveolarization
R² = 0.46,
p = 0.0002
CD68⁺ cells/mm² (lung)
% vWF area (HC, brain)
R² = 0.28,
p = 0.0054
Pdgfa fold change (lung)
% vWF area (HC, brain)
R² = 0.4,
p = 0.0005
Sftpc fold change (lung)
% vWF area (HC, brain)
R² = 0.54,
p < 0.0001
Vegfa fold change (lung)
% vWF area (HC, brain)

Figure S8: Myelination, microglia activation and vascularisation in the brain correlate with alveolarisation and septation markers in the lung. Brains and lungs of hyperoxia- or normoxia-exposed animals were analysed at P11. Gene and protein expression (sum pixel intensity) of MBP were correlated with the gene expression of the alveolarisation factor *Sftpc* and the mesenchymal marker *Pdgfrb* (A). Microglial activation in the brain (% CD68/IBA1 area) was correlated to gene expression levels of the alveolar factors *Aqp5* and *Pdgfrb* (B). Hippocampal vascularisation was correlated to macrophage infiltration (CD68 cell/mm²) and gene expression of the alveolarisation factors *Sftpc* and *Pdgfa* and the angiogenesis factor *Vegfa* (C). Correlation analyses are presented with a 95% confidence interval (grey area) and coefficient of determination (R^2). X-axes: brain values; Y-axes: lung values; black points: normoxia group; blue points: hyperoxia group.

Table S1: Antibodies used for immunohistochemistry on brain and lung tissue sections.

Antigen	dilution	host	supplier (catalogue number)
**CC1**	1:100	mouse	Calbiochem (OP80)
**CD68**	1:100	mouse	Bio-Rad (MCA341R)
**IBA1**	1:1000	rabbit	Wako (019-19741)
**MBP**	1:200	rat	Abcam (ab7349)
**OLIG2**	1:100	rabbit	Millipore (AB9610)
**OLIG2***	1:100	mouse	Millipore (MABN50)
**PDGFRA**	1:100	rabbit	Cell Signaling (3164S)
**vWF**	1:200	rabbit	Millipore (AB7356)

*Antibody used for co-staining with PDGFRA.

Table S2: Antibodies used for western blot analysis of brain samples.

Antigen	blocking	dilution	supplier (catalogue number)	sec. antibody (supplier, catalogue number)	dilution (sec. antibody)
**CNPase**	5% MP/TBS-T	1:2000	Millipore (MAB326)	anti-mouse IgG 1 (Novus Biologicals, NB7511)	1:5000
**IBA1**	5% MP/TBS-T	1:2000	Wako (016-200001)	anti-rabbit (Cell Signaling, #7074)	1:2000
**MAG**	5% MP/TBS-T	1:2000	Abcam (ab89780)	anti-mouse IgG 1 (Novus Biologicals, NB7511)	1:5000
**MBP**	5% MP/TBS-T	1:1000	Covance/ BioLegend (SMI99)	anti-mouse IgG 2b (Novus Biologicals, NB7521)	1:5000
**NG2**	5% BSA/TBS-T	1:1000	Cell Signaling (4235)	anti-rabbit (Cell Signaling, #7074)	1:2000
**OLIG2**	5% MP/TBS-T	1:2000	Millipore (AB9610)	anti-rabbit (DAKO, P0449)	1:2000

Abbreviations: BSA = bovine serum albumin, MP = non-fat milk powder, TBS-T = 0.1% Tween20 in Tris-buffered saline

Table S3: TaqMan Assays used for mRNA expression analysis in brain and lung tissues.

Gene	Assay ID
Acta2 *	Rn01759928
Aqp5 *	Rn00562837_m1
Arg1	Rn00691090_m1
B2m **	Rn00560865
Cd206	Rn01487342_m1
Cd86	Rn00571654_m1
Cnpase	Rn01399463_m1
Il-12	Rn00584538_m1
Mag	Rn01457782
Mbp	Rn01399619_m1
Ng2	Rn00578849
Olig2	Rn01767116
Pdgfa *	Rn00709363
Pdgfra *	Rn01399472
Pdgfrb *	Rn00709573
Pecam/Cd31 *	Rn01467262
Sftpc *	Rn00569225_m1
Vegfa **	Rn01511602

*Primers used on lung mRNA samples.
**Primers used on brain and lung mRNA samples.
